# Coronary artery bypass grafting-related bleeding complications in patients treated with ticagrelor or clopidogrel: a nationwide study

**DOI:** 10.1093/eurheartj/ehv381

**Published:** 2015-09-01

**Authors:** Emma C. Hansson, Lena Jidéus, Bengt Åberg, Henrik Bjursten, Mats Dreifaldt, Anders Holmgren, Torbjörn Ivert, Shahab Nozohoor, Mikael Barbu, Rolf Svedjeholm, Anders Jeppsson

**Affiliations:** 1 Department of Cardiothoracic Surgery, Sahlgrenska University Hospital, SE-413 45 Gothenburg, Sweden; 2 Department of Cardiothoracic Surgery, University Hospital, Uppsala, Sweden; 3 Department of Cardiothoracic Surgery, Blekinge Hospital, Karlskrona, Sweden; 4 Department of Cardiothoracic Surgery, Skåne University Hospital, Lund, Sweden; 5 Department of Cardiothoracic Surgery, University Hospital and University Health Care Research Centre, Örebro, Sweden; 6 Department of Cardiothoracic Surgery, University Hospital, Umeå, Sweden; 7 Karolinska University Hospital and Department of Molecular Medicine and Surgery, Karolinska Institutet, Stockholm, Sweden; 8 Department of Cardiothoracic Surgery, University Hospital, Linköping, Sweden; 9 Department of Molecular and Clinical Medicine, Institute of Medicine, The Sahlgrenska Academy, University of Gothenburg, Gothenburg, Sweden

**Keywords:** Dual antiplatelet therapy, Acute coronary syndrome, Bleeding complications, Cardiac surgery

## Abstract

**Aims:**

Excessive bleeding impairs outcome after coronary artery bypass grafting (CABG). Current guidelines recommend withdrawal of clopidogrel and ticagrelor 5 days (120 h) before elective surgery. Shorter discontinuation would reduce the risk of thrombotic events and save hospital resources, but may increase the risk of bleeding. We investigated whether a shorter discontinuation time before surgery increased the incidence of CABG-related major bleeding complications and compared ticagrelor- and clopidogrel-treated patients.

**Methods and results:**

All acute coronary syndrome patients in Sweden on dual antiplatelet therapy with aspirin and ticagrelor (*n* = 1266) or clopidogrel (*n* = 978) who underwent CABG during 2012–13 were included in a retrospective observational study. The incidence of major bleeding complications according to the Bleeding Academic Research Consortium-CABG definition was 38 and 31%, respectively, when ticagrelor/clopidogrel was discontinued <24 h before surgery. Within the ticagrelor group, there was no significant difference between discontinuation 72–120 or >120 h before surgery [odds ratio (OR) 0.93 (95% confidence interval, CI, 0.53–1.64), *P* = 0.80]. In contrast, clopidogrel-treated patients had a higher incidence when discontinued 72–120 vs. >120 h before surgery (OR 1.71 (95% CI 1.04–2.79), *P* = 0.033). The overall incidence of major bleeding complications was lower with ticagrelor [12.9 vs. 17.6%, adjusted OR 0.72 (95% CI 0.56–0.92), *P* = 0.012].

**Conclusion:**

The incidence of CABG-related major bleeding was high when ticagrelor/clopidogrel was discontinued <24 h before surgery. Discontinuation 3 days before surgery, as opposed to 5 days, did not increase the incidence of major bleeding complications with ticagrelor, but increased the risk with clopidogrel. The overall risk of major CABG-related bleeding complications was lower with ticagrelor than with clopidogrel.

**See page 198 for the editorial comment on this article (doi:10.1093/eurheartj/ehv469)**

## Introduction

Dual antiplatelet therapy (DAPT) with acetylsalicylic acid and a P2Y_12_-receptor antagonist reduces the risk of thrombotic complications compared with treatment with only acetylsalicylic acid in patients with acute coronary syndrome (ACS).^1^ The risk of thrombotic complications is further reduced if one of the new more potent platelet inhibitors, ticagrelor or prasugrel, is used instead of clopidogrel,^2,3^ but the risk of both spontaneous and surgical bleeding complications may increase with the new inhibitors.^3,4^

Major bleeding complications impair outcome after cardiac surgery.^5,6^ Acute coronary syndrome patients on DAPT who need acute or urgent coronary artery bypass grafting (CABG) are at high risk of major bleeding.^6–8^ Current revascularization guidelines therefore recommend that clopidogrel and ticagrelor are discontinued 5 days before surgery and prasugrel 7 days before elective surgery,^9,10^ but the patient's condition may render this impossible. Most ACS patients are hospitalized while waiting for CABG. If a shorter discontinuation time of the platelet inhibitor would be safe from a bleeding perspective, it would reduce the risk of thrombotic complications during the waiting time, and save hospital resources. The risk of bleeding complications in relation to discontinuation time has not previously been investigated in real life in sufficiently large patient cohorts.

The newer and more potent platelet inhibitors may increase the risk of major CABG-related bleeding complications, but it is unclear whether the incidence differs between ticagrelor and clopidogrel in real life after adjustment for time since discontinuation and other factors that influence bleeding risk.

The aims of the present real-life study were to investigate whether a shorter discontinuation time before surgery increases the risk of major bleeding with ticagrelor or clopidogrel and to compare the unadjusted and adjusted incidence of CABG-related bleeding complications between clopidogrel and ticagrelor.

## Methods

### Study patients

The study was a retrospective analysis of prospectively collected data. All 2244 ACS patients on DAPT who underwent acute or urgent CABG in Sweden from January 2012 to December 2013 were included in a retrospective study. The patients were treated preoperatively with acetylsalicylic acid (aspirin) and either ticagrelor (*n* = 1266) or clopidogrel (*n* = 978) within the last 14 days before surgery. In 2012–13, ticagrelor was introduced in the Swedish regional guidelines to replace clopidogrel as the first treatment option in ACS patients planned for interventional treatment. Prasugrel is also used in Sweden. Patients treated with prasugrel were included in the registry but not in this analysis, due to the small number, just 10 patients, over the study period. The patients underwent CABG at one of the eight cardiothoracic surgery centres in Sweden: Umeå University Hospital (*n* = 291), Uppsala University Hospital (*n* = 97), Karolinska University Hospital (*n* = 267), Örebro University Hospital (*n* = 90), Linköping University Hospital (*n* = 326), Sahlgrenska University Hospital (*n* = 473), Blekinge Hospital (*n* = 130), and Skåne University Hospital, Lund (*n* = 570). The study was conducted in accordance with the Declaration of Helsinki, and was approved by the Regional Research Ethics Committee in Gothenburg on 30 April 2014 (reference number 031-14), which waived the need for individual consent from the patients before inclusion in the registry. Preoperative patient characteristics are summarized in *Table 1*.

**Table 1:** Baseline demographics and preoperative variables

	Clopidogrel (*n* = 978)	Ticagrelor (*n* = 1266)	*P*-value
Female gender	203 (20.8%)	271 (21.4%)	0.75
Age (years)	68.4 ± 9.5 *n* = 978	67.8 ± 9.4 *n* = 1266	0.082
BMI (kg/m^2^)	27.3 ± 4.2 *n* = 976	27.3 ± 4.0 *n* = 1262	0.38
Diabetes	252 (25.8%)	347 (27.4%)	0.44
Preoperative haemoglobin (g/L)	137 ± 16 *n* = 978	136 ± 15 *n* = 1266	0.068
Preoperative platelet count (10^9^/L)	246 ± 73 *n* = 965	250 ± 73 *n* = 1255	0.066
Preoperative creatinine (μmol/L)	95 ± 72 *n* = 975	91 ± 42 *n* = 1259	0.86
Preoperative prothrombin time (INR)	1.09 ± 0.30 *n* = 958	1.08 ± 0.16 *n* = 1242	0.97
Preoperative APTT (s)	36 ± 19 *n* = 878	36 ± 18 *n* = 1163	0.0056
EuroSCORE I (additive)	Mean 5.62 ± 3.28 Median 5.0 (0.0–20) *n* = 974	Mean 5.50 ± 3.14 Median 5.0 (0.0–22) *n* = 1254	0.49
Ejection fraction (%)			
>50	607 (62.4%)	792 (63.4%)	0.14
31–50	290 (29.8%)	392 (31.4%)	
>20–30	66 (6.8%)	60 (4.8%)	
≤20	10 (1.0%)	6 (0.5%)	
Warfarin treatment at any time before surgery	47 (4.8%)	26 (2.1%)	0.0005
Fondaparinux at any time before surgery	645 (66.2%)	919 (72.6%)	0.0011
LMWH at any time before surgery	221 (22.6%)	373 (29.6%)	0.0002
GPIIb/IIIa inhibitor before surgery	2 (0.2%)	3 (0.2%)	1.0
Discontinuation of clopidogrel/ticagrelor (days)	5.2 ± 3.6 Median 5 (0–14)	5.9 ± 3.5 Median 6 (0–14)	<0.0001
Discontinuation (h)			
0–24	65 (6.6%)	110 (8.7%)	<0.0001
24–48	147 (15.0%)	62 (4.9%)	
48–72	76 (7.8%)	54 (4.3%)	
72–96	71 (7.3%)	89 (7.0%)	
96–120	73 (7.5%)	104 (8.2%)	
>120	546 (55.8%)	847 (66.9%)	
Acute surgery	99 (10.1%)	159 (12.6%)	0.080

Values are given as mean ± SD, median (interval), or frequency (percent). *P*-values from Fisher's exact test for dichotomous variables, Mantel–Haenszel *χ*^2^ test for ordered categorical variables, and Mann–Whitney *U*-test for continuous variables.

BMI, body mass index; INR, international normalized ratio; APTT, activated partial thromboplastin time; LMWH, low-molecular-weight heparin; GPIIb/IIIa, glycoprotein IIb/IIIa.

### Study design

The patients were identified in the SWEDEHEART registry^11^ and/or institutional databases. Data were obtained from SWEDEHEART, hospital records, and the participating hospitals' surgical databases, and were compiled in a nationwide registry. The patients were grouped according to the platelet inhibitor used. If a patient had been treated with both agents, the patient was classified according to the last medication before surgery. Outcome variables assessed were incidence of major bleeding complications in the clopidogrel and ticagrelor groups, overall and after adjustment. We also compared the incidence of major bleeding complications within and between the ticagrelor and clopidogrel groups when the platelet inhibitor was discontinued 0–72, 72–120, or >120 h before surgery, assessed postoperative bleeding volume during the first 12 postoperative hours in relation to the timing of discontinuation of the platelet inhibitor, and the incidence and number of allogeneic blood products [red blood cells (RBCs), plasma, and platelets] transfused during the index hospital stay in relation to the period of discontinuation. Thirty-day mortality and thrombotic events during hospital stay were only registered for safety reasons. No statistical testing or detailed analysis was performed for these variables, due to the lack of statistical power.

### Definitions

Major bleeding was defined according to four published definitions: Bleeding Academic Research Consortium (BARC) type 4, CABG-related bleeding (bleeding resulting in death, or reoperation due to bleeding, or intracranial haemorrhage, or transfusion of 5 or more units of RBCs over 48 h, or chest tube drainage in excess of 2000 mL over 24 h),^12^ Blood Conservation Using Antifibrinolytics in a Randomized Trial (BART; postoperative blood loss >1500 mL/12 h, or re-exploration due to bleeding, or RBC transfusion of 10 units or more, or death because of bleeding),^13^ PLATelet inhibition and patient Outcomes (PLATO) life-threatening bleeding (fatal bleeding, or pericardial bleeding requiring repeat surgery, or drop in haemoglobin of ≥50 g/L, or transfusion of 4 or more units of RBCs), and PLATO major bleeding (pericardial bleeding requiring repeat surgery, or drop in haemoglobin of ≥30 g/L, or transfusion of 2 or more units of RBCs).^2^

Major bleeding is reported for all four bleeding definitions while adjusted data are reported only for the BARC-CABG definition. Bleeding volume was defined as mediastinal drainage volume. A thrombotic event was defined as ischaemic stroke with duration exceeding 24 h and verified by CT or MRI, pulmonary embolism, or deep vein thrombosis evidenced during the index hospitalization. Acute surgery was defined as procedure starting within 24 h of acceptance. Urgent surgery was defined as within hospitalization for ACS.

### Clinical management

Patients were treated in accordance with standard practice at the participating centres. All patients received 75 mg aspirin daily. Ticagrelor or clopidogrel was administered with a loading dose followed by 75 mg once daily for clopidogrel or 90 mg twice daily for ticagrelor. According to the current European guidelines, the platelet inhibitor should be discontinued 5 days prior to surgery if clinically feasible.^9^ Fondaparinux and low-molecular-weight heparin (LMWH) were discontinued at least 12 h before non-acute surgery. Aspirin was not discontinued before surgery.

### Statistical analysis

The two groups were compared at baseline by Fisher's exact test for dichotomous variables, the Mantel–Haenszel *χ*^2^ test for ordered categorical variables, and the Mann–Whitney *U*-test for continuous variables. Logistic regression modelling was used to identify factors related to major bleeding and to compare incidence of bleeding between discontinuation groups. Factors that were significantly different between platelet inhibitors and associated with major bleeding with a *P*-value of <0.10 were included in a multivariable logistic regression model for adjustment.

The sample size (at least 500 patients in each group) was chosen to achieve 80% power in finding a significant difference in the incidence of major bleeding complications between clopidogrel and ticagrelor after stratification by time from discontinuation of medication to surgery. The power estimation was based on a previous single-centre study.^6^ Data are presented as mean (± standard deviation), median (range) or frequency (percent). Statistical signiﬁcance was assumed with a two-sided *P*-value of <0.05. No adjustment for multiplicity was performed. SAS software, version 9.4 (SAS Institute, Cary, NC, USA), was used for statistical analysis.

## Results

### Baseline variables

Demographic data are presented in *Table 1*. More clopidogrel-treated patients were treated with warfarin at some time prior to surgery, 4.8 vs. 2.1% (*P* = 0.0005). Only 13 patients, 5 in the ticagrelor group and 8 in the clopidogrel group, were treated with warfarin <5 days before surgery. The preoperative prothrombin time did not differ between groups. Preoperative treatment with LMWH and fondaparinux was more common in the ticagrelor group. In 44.2% of the clopidogrel-treated patients, the platelet inhibitor was discontinued <5 days prior to surgery, compared with 33.1% in the ticagrelor group (*P* < 0.0001), and mean discontinuation was 5.2 ± 3.6 days for the clopidogrel-treated patients compared with 5.9 ± 3.5 days for the ticagrelor-treated patients (*P* < 0.0001).

### Procedures

All but 16 of the patients (99.3%) were operated with cardiopulmonary bypass, and mean cardiopulmonary bypass time was marginally longer in the clopidogrel group (81 ± 37 vs. 77 ± 31 min; *P* = 0.025). Total operation time was not significantly different between clopidogrel and ticagrelor (193 ± 67 vs. 189 ± 57 min; *P* = 0.66) and neither was duration of aortic cross clamp (47 ± 22 vs. 46 ± 20 min; *P* = 0.23). The number of distal anastomoses did not differ between the ticagrelor group and the clopidogrel group (3.2 ± 1.0 vs. 3.3 ± 1.0, *P* = 0.12), and concomitant valve repair was performed in 2.0% of cases for clopidogrel and 2.5% for ticagrelor (*P* = 0.54).

**Table 2:** Postoperative outcome variables

	Clopidogrel (*n* = 978)	Ticagrelor (*n* = 1266)	*P*-value
Postoperative bleeding (mL)			
12 h	Mean 614 ± 393 Median 500 (0–3940)	Mean 579 ± 411 Median 470 (100–5475)	0.0017
24 h	Mean 830 ± 498 Median 700 (0–5398)	Mean 813 ± 554 Median 671 (140–7501)	0.093
Incidence of transfusion			
Any	559 (57.2%)	645 (51.0%)	0.0038
RBC	517 (52.9%)	589 (46.6%)	0.0028
Plasma	238 (24.4%)	240 (19.0%)	0.0025
Platelets	226 (23.1%)	263 (20.8%)	0.20
Amount of transfusions (U)			
Any	Mean 3.95 ± 7.25 Median 2 (0–72)	Mean 3.92 ± 10.18 Median 1 (0–176)	0.0012
RBC	Mean 2.42 ± 3.92 Median 1 (0–41)	Mean 2.26 ± 4.93 Median 0 (0–73)	0.0012
Plasma	Mean 1.01 ± 2.93 Median 0 (0–36)	Mean 1.01 ± 4.11 Median 0 (0–79)	0.0036
Platelets	Mean 0.525 ± 1.271 Median 0 (0–12)	Mean 0.653 ± 1.869 Median 0 (0–24)	0.43
Reoperation due to bleeding	74 (7.6%)	77 (6.1%)	0.19
Lowest postoperative haemoglobin (g/L)	Mean 92 ± 12 Median 91 (51–147)	Mean 93 ± 12 Median 92 (52–150)	0.018
Highest postoperative creatinine (μmol/L)	Mean 115 ± 81 Median 94 (42–980)	Mean 112 ± 75 Median 91 (40–1055)	0.082
Time in ICU (days)	Mean 2.26 ± 3.37 Median 1.0 (0–41)	Mean 2.0 ± 3.04 Median 1.0 (0–38)	0.0008
Hospital length-of-stay after CABG (days)	Mean 7.83 ± 5.11 Median 7.0 (1–62)	Mean 7.58 ± 5.20 Median 6.0 (1–69)	0.0005

Values are given as mean ± SD, median (interval), or frequency (percent). *P*-values from Fisher's exact test for dichotomous variables, Mantel–Haenszel *χ*^2^ test for ordered categorical variables, and Mann–Whitney *U*-test for continuous variables.

RBC, red blood cells; ICU, intensive care unit; CABG, coronary artery bypass grafting.

### Major bleeding

**Figure 1:**
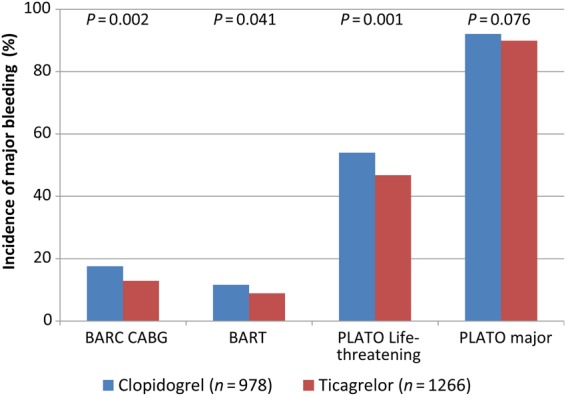
Incidence of major bleeding complications according to BARC-CABG, BART, PLATO life-threatening, and PLATO major bleeding (*P*-values from Fisher's exact test between ticagrelor and clopidogrel). BARC, Bleeding Academic Research Consortium; CABG, coronary artery bypass grafting; BART, Blood Conservation Using Antifibrinolytics in a Randomized Trial; PLATO, PLATelet inhibition and patient Outcomes.

**Figure 2:**
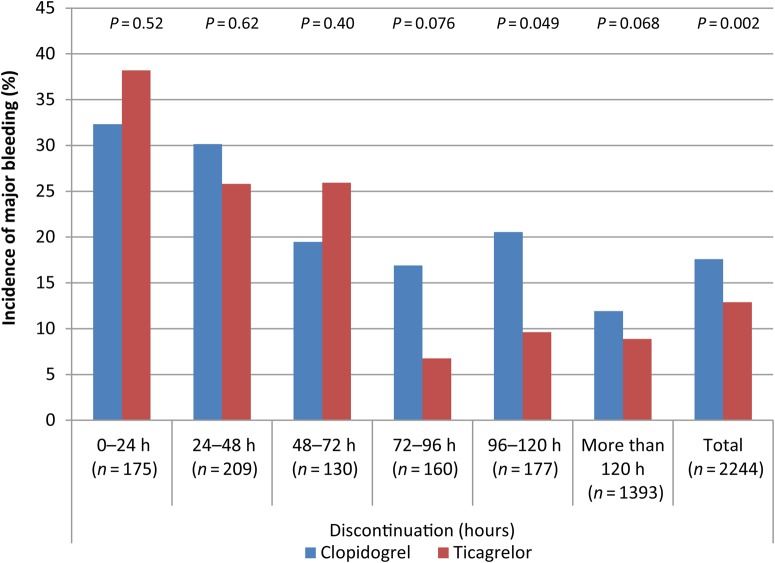
Incidence of BARC-CABG major bleeding by day of discontinuation of clopidogrel/ticagrelor to surgery (*P*-values from Fisher's exact test). BARC, Bleeding Academic Research Consortium; CABG, coronary artery bypass grafting.

Overall, there were significantly less CABG-related major bleeding complications in ticagrelor-treated patients according to three of the four definitions (*Figure 1*): BARC-CABG 12.9 vs. 17.6% (*P* = 0.0024), BART major bleeding 8.8 vs. 11.6% (*P* = 0.041), and PLATO life-threatening major bleeding 46.8 vs. 54.0% (*P* = 0.0008) for ticagrelor- and clopidogrel-treated patients, respectively. PLATO major bleeding did not differ significantly (89.9 vs. 92.1%; *P* = 0.076). Incidence of BARC-CABG major bleeding by day of discontinuation is shown in *Figure 2*. Other postoperative outcome variables are summarized in *Table 2*.

The use of ticagrelor was associated with a reduced risk of BARC-CABG major bleeding both before adjustment [odds ratio (OR) 0.69 (95% confidence interval, CI, 0.55–0.87), *P* = 0.002] and after adjustment for time since discontinuation and the other factors significantly influencing the risk of bleeding in the univariable analysis [adjusted OR 0.72 (95% CI 0.56–0.92), *P* = 0.012]. Other factors associated with major bleeding in univariable logistic regression are presented in *Table 3*.

**Table 3:** Factors associated with major bleeding in univariable logistic regression

	No major bleeding (*n* = 1909)	Major bleeding (*n* = 335)	Unadjusted OR (95% CI)	*P*-value
Female gender	20%	27.8%	1.54 (1.18–2.01)	0.0013
Age (years, OR per 10 years)	67.8 ± 9.3	69.9 ± 9.9	1.28 (1.12–1.46)	0.0002
BMI (kg/m^2^)	27.4 ± 4.1	26.9 ± 4.1	0.97 (0.94–1.00)	0.0541
Diabetes	26.7%	26.9%	1.01 (0.78–1.32)	0.9225
Preoperative haemoglobin (g/L, OR per 10 units)	138 ± 15	132 ± 17	0.80 (0.74–0.86)	<0.0001
Preoperative platelet count (10^9^/L, OR per 10 units)	247 ± 71	257 ± 82	1.02 (1.00–1.03)	0.0156
Preoperative creatinine (μmol/L, OR per 10 units)	90 ± 52	104 ± 83	1.03 (1.01–1.05)	0.0004
Preoperative prothrombin time (INR)	1.1 ± 0.2	1.1 ± 0.1	1.10 (0.71–1.71)	0.6582
Preoperative APTT (s, OR per 10 units)	35 ± 16	41 ± 27	1.13 (1.07–1.19)	<0.0001
EuroSCORE I (additive)	5.2 ± 2.9	7.3 ± 4.1	1.20 (1.16–1.24)	<0.0001
Ejection fraction (%)				
>50	66.7%	49.2%	1.84 (1.56–2.17)	<0.0001
31–50	30.1%	37.7%		
20–30	2.6%	11.2%		
≤20	0.5%	1.9%		
Platelet inhibitor (ticagrelor)	57.8%	48.7%	0.69 (0.55–0.87)	0.0020
Warfarin treatment	3.1%	4.5%	1.49 (0.83–2.66)	0.1796
Fondaparinux treatment	70.3%	67.2%	0.86 (0.67–1.11)	0.2509
Heparin treatment	14.8%	22.2%	1.65 (1.23–2.19)	0.0007
LMWH treatment	27.2%	22.8%	0.79 (0.60–1.04)	0.0889
Operation duration (min, OR per h)	183 ± 49	231 ± 99	1.92 (1.71–2.15)	<0.0001
CPB duration (min, OR per h)	76 ± 30	95 ± 48	2.37 (1.95–2.88)	<0.0001
Cross-clamp duration (min, OR per h)	46 ± 19	51 ± 29	1.88 (1.38–2.55)	<0.0001
Concomitant valve proc.	1.6%	5.7%	3.84 (2.14–6.91)	<0.0001
Acute surgery	8.8%	27.2%	3.89 (2.92–5.19)	<0.0001
Discontinuation of platelet inhibitor (days)	5.9 ± 3.5	3.9 ± 3.5	0.84 (0.81–0.87)	<0.0001

Odds ratio (OR) per increase of one unit of the predictor if not otherwise indicated.

BMI, body mass index; INR, international normalized ratio; APTT, activated partial thromboplastin time; LMWH, low-molecular-weight heparin; CPB, cardiopulmonary bypass.

### Bleeding volume and transfusions

Overall, ticagrelor-treated patients bled less after surgery, and received fewer transfusions of blood products (*Table 2*). However, when medication was discontinued <24 h before surgery, ticagrelor-treated patients bled markedly more and received more transfusions than clopidogrel-treated patients (*Table 4*).

**Table 4:** Bleeding and transfusions by time from discontinuation of platelet inhibitor

	Discontinuation	Clopidogrel		Ticagrelor		*P*-value
		Mean ± SD	Median	Mean ± SD	Median	
Postoperative bleeding first 12 h (mL)	0–24 h	663 ± 627	488 (340–721)	813 ± 478	670 (498–1103)	<0.001
	24–48 h	714 ± 462	600 (415–890)	641 ± 337	585 (400–753)	0.514
	48–72 h	659 ± 313	570 (440–815)	709 ± 707	510 (370–735)	0.207
	72–96 h	682 ± 462	560 (400–790)	630 ± 541	450 (343–738)	0.118
	96–120 h	701 ± 454	520 (405–800)	550 ± 296	450 (350–698)	0.036
	Over 120 h	555 ± 313	480 (358–653)	534 ± 363	450 (349–610)	0.021
Any transfusion (units)	0–24 h	8.9 ± 13.4	4 (2–11)	14.7 ± 22.5	8.5 (4–17)	0.001
	24–48 h	5.8 ± 8.6	3 (0–8)	7.9 ± 9.8	4 (2–11)	0.111
	48–72 h	4.9 ± 6.4	3 (0–6)	7.6 ± 17.4	3 (0–7)	0.779
	72–96 h	4.6 ± 8.7	2 (0–6)	2.6 ± 4.7	2 (0–3)	0.013
	96–120 h	3.6 ± 4.7	2 (0–6)	2.0 ± 3.7	0 (0–3)	0.025
	Over 120 h	2.7 ± 5.4	0 (0–3)	2.4 ± 6.3	0 (0–2)	0.044
RBC transfusion (units)	0–24 h	4.9 ± 6.8	2 (1–6)	6.9 ± 9.8	4.5 (2–9)	0.028
	24–48 h	3.4 ± 4.5	2 (0–5)	4.4 ± 5.7	2 (0–6.3)	0.400
	48–72 h	2.8 ± 3.5	2 (0–3.8)	4.0 ± 9.9	2 (0–4)	0.618
	72–96 h	3.0 ± 5.3	2 (0–4)	1.7 ± 3.2	0 (0–2)	0.033
	96–120 h	2.3 ± 2.9	1 (0–4)	1.3 ± 2.1	0 (0–2)	0.046
	Over 120 h	1.7 ± 3.0	0 (0–3)	1.6 ± 3.2	0 (0–2)	0.096
Plasma transfusion (units)	0–24 h	2.5 ± 5.5	0 (0–3)	4.6 ± 10.1	2 (0–4.3)	0.022
	24–48 h	1.5 ± 3.6	0 (0–2)	1.9 ± 3.2	0 (0–2.3)	0.154
	48–72 h	1.3 ± 2.7	0 (0–1.8)	1.8 ± 4.9	0 (0–1)	0.480
	72–96 h	0.96 ± 2.8	0 (0–0)	0.54 ± 1.3	0 (0–0)	0.490
	96–120 h	0.84 ± 1.6	0 (0–1)	0.35 ± 1.2	0 (0–0)	0.001
	Over 120 h	0.70 ± 2.4	0 (0–0)	0.56 ± 2.7	0 (0–0)	0.005
Platelet transfusion (units)	0–24 h	1.5 ± 2.3	0 (0–2)	3.2 ± 3.7	2 (0.8–4)	<0.001
	24–48 h	0.94 ± 1.5	0 (0–2)	1.6 ± 2.2	1 (0–2)	0.033
	48–72 h	0.79 ± 1.4	0 (0–1)	1.8 ± 3.7	0 (0–2)	0.430
	72–96 h	0.68 ± 1.4	0 (0–1)	0.44 ± 0.81	0 (0–0.5)	0.563
	96–120 h	0.51 ± 1.0	0 (0–1)	0.32 ± 0.90	0 (0–0)	0.086
	Over 120 h	0.25 ± 0.84	0 (0–0)	0.24 ± 0.95	0 (0–0)	0.357

Values are given as mean ± SD and median (25–75 percentiles). For comparison between groups, the Mann–Whitney *U*-test was used.

RBC, red blood cells; SD, standard deviation.

### Impact of time since discontinuation

**Figure 3:**
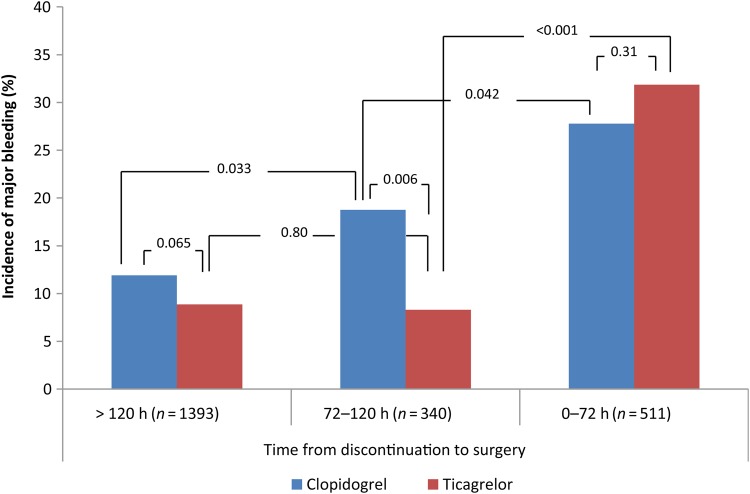
Incidence of BARC-CABG major bleeding stratified by time from discontinuation of clopidogrel/ticagrelor to surgery (*P*-values denoting difference between the platelet inhibitors, and within clopidogrel/ticagrelor between discontinuation strata as indicated). BARC, Bleeding Academic Research Consortium; CABG, coronary artery bypass grafting.

The difference in the incidence of major bleeding complications between ticagrelor and clopidogrel was mainly driven by a significant reduction in major bleeding complications in the ticagrelor group when clopidogrel/ticagrelor was discontinued 72–120 h before surgery [unadjusted OR 0.39 (95% CI 0.20–0.76), *P* = 0.006, *Figure 3*]. When either drug was discontinued according to the current guidelines (>120 h before surgery), there was no significant difference in the incidence of major bleeding complications between ticagrelor- and clopidogrel-treated patients [9 vs. 12%; unadjusted OR 0.72 (95% CI 0.51–1.02), *P* = 0.065].

Within the ticagrelor group, there was no significant difference in major bleeding complications between discontinuation 72–120 or >120 h before surgery [unadjusted OR 0.93 (95% CI 0.53–1.64), *P* = 0.80], whereas discontinuation 0–72 h was associated with a significantly higher rate of major bleeding compared with both 72–120 h [unadjusted OR 5.17 (95% CI 2.89–9.27), *P* < 0.0001] and >120 h [unadjusted OR 4.81 (95% CI 3.34–6.95), *P* < 0.0001]. In contrast, clopidogrel-treated patients had a higher incidence of major bleeding complications when discontinued 72–120 compared with >120 h before surgery [unadjusted OR 1.71 (95% CI 1.04–2.79), *P* = 0.033]. Likewise, in the clopidogrel group, discontinuation 0–72 h was associated with an increased incidence of major bleeding compared with 72–120 h [unadjusted OR 1.67 (95% CI 1.02–2.73), *P* = 0.042] and >120 h [unadjusted OR 2.85 (95% CI 1.98–4.10), *P* < 0.0001] (*Figure 3*).

### Mortality and thrombotic events

Thirty-day mortality was 1.7% in the ticagrelor group and 2.7% in the clopidogrel group. The mortality was significantly higher in patients with major bleeding complications [9.9 vs. 0.7%, unadjusted OR 14.78 (95% CI 7.82–27.93), *P* < 0.0001]. Thrombotic events during the postoperative hospital stay occurred in 2.3% of the ticagrelor group and 2.8% of the clopidogrel group.

## Discussion

The main finding of this nationwide registry study was that discontinuation of the platelet inhibitor 3 days before surgery, as opposed to 5 days, did not increase the incidence of major bleeding complications in ticagrelor-treated patients, but increased the incidence in clopidogrel-treated patients. In addition, a lower incidence of major bleeding complications was observed in ticagrelor-treated patients, except when the platelet inhibitor was discontinued <72 h before surgery.

The timing of discontinuation of the platelet inhibitor is influenced by a number of factors, including institutional guidelines, the patient's condition, logistical reasons, and the individual surgeon's decision. This is illustrated in the present study where 45% of the clopidogrel patients and 33% of the ticagrelor patients were operated after a shorter discontinuation than the 5 days recommended in the guidelines. We used this variation to compare the incidence of major bleeding complications after different discontinuation times.

The data in the present study were collected during 2012 and 2013. During this period, ticagrelor replaced clopidogrel as first-choice P2Y_12_-receptor antagonist for ACS patients in the Swedish guidelines. The introduction of ticagrelor did not occur simultaneously in all parts of Sweden; instead, there was a gradual increase in the use of ticagrelor over time. Consequently, during this time period, there were ACS patients with the same characteristics treated with either ticagrelor or clopidogrel. We deliberately used this time period for the study to obtain comparable groups.

The most clinically important observation in this study was the lack of difference in major bleeding complications when ticagrelor was discontinued 3 days before surgery compared with 5 days. This suggests that it is safe to operate on ACS patients treated with ticagrelor earlier after discontinuation than is currently recommended in guidelines. A reduction in the waiting time from 5 to 3 days would reduce the risk for thrombotic events while waiting for CABG, and save hospital resources.

The results of the study also confirm the detrimental effect of major bleeding complications on outcome after CABG. The unadjusted 30-day mortality was almost 15 times greater in patients with major bleeding compared with those without bleeding complications (9.9 vs. 0.7%). This corroborates previous registry studies about the effects of bleeding complications^5,6,14,15^ and emphasizes the importance of, if possible, avoiding excessive bleeding after CABG. Timely discontinuation of platelet inhibitors would help to achieve this.

We also observed a lower overall incidence of major bleeding complications in patients treated with ticagrelor. It should be pointed out that the result in this regard is hypothesis-generating rather than conclusive, given the retrospective observational design of the study. The lower incidence of major bleeding complications in the ticagrelor group in the present study could not be explained by the longer discontinuation time in the ticagrelor group (*Table 1*), since the difference was maintained also after adjustment. Instead, it is mainly explained by the lower incidence of major bleeding complications in the ticagrelor group when ticagrelor/clopidogrel was discontinued 3–5 days before surgery. This is, in turn, explained by differences in the pharmacokinetic and pharmacodynamic profiles of ticagrelor and clopidogrel. Ticagrelor is a direct-acting P2Y_12_-receptor antagonist with greater antiplatelet effect and more consistent platelet inhibition than clopidogrel.^16,17^ Ticagrelor has also faster on-set of action (within 30 min of loading) and faster off-set, i.e. the antiplatelet effect of ticagrelor returns faster to baseline than with clopidogrel.^16^ Despite these known difference in off-set time of the antiplatelet effect, current guidelines recommend that both ticagrelor and clopidogrel are discontinued 5 days before surgery.^9,10^ The results of the present study instead support the use of differentiated discontinuation times for ticagrelor and clopidogrel, i.e. 3 days for ticagrelor and 5 days for clopidogrel.

There was a higher incidence of transfusions and a larger bleeding volume in the ticagrelor group when the platelet inhibitor was discontinued within 24 h before surgery (*Table 4*), even if the difference in the incidence of major bleeding complications according to the BARC-CABG definition did not reach statistical significance (38 vs. 31%). This indicates, in accordance with previous studies,^6,18^ that the risk for severe bleeding is higher with ticagrelor than with clopidogrel if it cannot be discontinued before surgery, which also is consistent with the stronger antiplatelet effect of ticagrelor compared with clopidogrel.^16,17^ This increased bleeding risk with ticagrelor may be considered before loading if there is high risk for acute CABG. In contrast, there was no significant difference in bleeding complications in the present study when clopidogrel and ticagrelor were discontinued in accordance with the current guidelines, i.e. 5 days or more before CABG.

The incidence of major bleeding complications was higher in women than in men (*Table 3*). This may at least partly be due to the higher risk of transfusions in women, since transfusions are included in all definitions of major bleeding utilized in the present study. Postoperative bleeding volume was not larger in women than in men in this material (data not shown). The higher risk of transfusion in women is mainly caused by lower preoperative haemoglobin levels and smaller blood volume, leading to a higher grade of haemodilution during cardiopulmonary bypass.

Current guidelines recommend discontinuation of the platelet inhibitor at a fixed time interval before surgery. In the future, platelet function tests may be used to optimize timing of the procedure rather than the choice of drug and the corresponding interval. This strategy is supported by a recent study, where Ranucci *et al.*^19^ show a significant association between the grade of ADP-dependent platelet aggregability and CABG-related bleeding complications in clopidogrel-treated patients. So far, no data are available in ticagrelor-treated patients.

The present study has all inherent limitations of an observational study, including selection bias and unregistered confounders. Unregistered confounders may include, e.g. history of bleeding, liver disease, heart failure, and renal failure. To prove the current findings, a randomized clinical trial with different discontinuation times would be required. The study also has strengths; it represents a complete nationwide cohort of ACS patients on DAPT with ticagrelor or clopidogrel operated with acute or urgent CABG in Sweden during a 2-year period, giving a relatively large cohort of similar patients. The gradual introduction of the new inhibitor over the country gave an opportunity for comparison, even though the groups are not randomized.

In conclusion, discontinuation of ticagrelor 3 days before surgery did not increase the risk of major bleeding complications after CABG compared with 5 days. The overall risk of major CABG-related bleeding complications was lower with ticagrelor than with clopidogrel in this real-life observational study. The difference was driven by a lower incidence with ticagrelor than with clopidogrel when the platelet inhibitor was discontinued 72–120 h before surgery.

## Funding

This research was conducted with support from an Investigator Sponsored Study Programme of AstraZeneca and the Swedish Heart and Lung Foundation [Grant numbers: 20120372, 2014021]. The study sponsors had no influence on the analysis and interpretation of data, on the writing of the report, or on the decision to submit the paper for publication. Funding to pay the Open Access publication charges for this article was provided by AstraZeneca.

**Conflict of interest**: E.C.H. and A.J. have received speaker's honorarium from AstraZeneca. A.J. has also received support from AstraZeneca for other investigator-initiated studies.
